# Metabolomics Combined with Transcriptomics Analysis Revealed the Amino Acids, Phenolic Acids, and Flavonol Derivatives Biosynthesis Network in Developing *Rosa roxburghii* Fruit

**DOI:** 10.3390/foods11111639

**Published:** 2022-06-01

**Authors:** Nanyu Li, Lanlan Jiang, Yiyi Liu, Shimei Zou, Min Lu, Huaming An

**Affiliations:** 1 Agricultural College, Guizhou University, Guiyang 550025, China; liny4102022@163.com (N.L.); j17385313153@163.com (L.J.); lyylyy0428@163.com (Y.L.); shimei_zou@163.com (S.Z.); 2 National Forestry and Grassland Administration Engineering Research Center for *Rosa roxburghii*, Guiyang 550025, China

**Keywords:** *Rosa roxburghii* fruits, LC-MS/MS, transcriptome, amino acids, polyphenols, flavonoids

## Abstract

*Rosa roxburghii* Tratt. is a specific fruit with high nutritional value and antioxidative activities. However, the key metabolites and their biosynthesis are still unknown. Herein, a main cultivated variety, ‘Guinong 5’ (Rr5), was chosen to analyze the metabolomics of the three developmental stages of *R. roxburghii* fruit by liquid chromatography–tandem mass spectrometry (LC-MS/MS). A total of 533 metabolites were identified, of which 339 were significantly altered. Total phenols, flavonoids, and amino acids were significantly correlated to at least one in vitro antioxidant activity. The conjoint Kyoto Encyclopedia of Genes and Genomes (KEGG) co-enrichment analysis of metabolome and transcriptome was focused on amino acid, phenylpropanoid, and flavonoid biosynthesis pathways. The amino acid, phenolic acid, and flavonol biosynthesis networks were constructed with 32 structural genes, 48 *RrMYBs*, and 23 metabolites. Of these, six *RrMYBs* correlated to 9–15 metabolites in the network were selected to detect the gene expression in six different *R. roxburghii* genotypes fruits. Subsequently, 21 key metabolites were identified in the in vitro antioxidant activities in the fruits at various developmental stages or in fruits of different *R. roxburghii* genotypes. We found that four key *RrMYBs* were related to the significantly varied amino acids, phenolic acids, and flavonol derivatives in the network during fruit development and the key metabolites in the in vitro antioxidative activities in the fruits of six *R. roxburghii* genotypes. This finding provided novel insights into the flavonoid, polyphenol, and amino acid synthesis in *R. roxburghii*.

## 1. Introduction

*Rosa roxburghii* Tratt. is a newly popular rosaceous plant with uniquely flavored fruits and a high nutritional and medical value. The fruits are rich in L-ascorbic acid (AsA) and organic acids, amino acids, phenols, flavonoids, superoxide dismutase (SOD), and triterpenoids, among which total phenols, flavonoids, AsA, and triterpenoids contribute >80% to the antioxidant activity [1,2,3,4,5]. Jiang et al. identified 723 metabolites in six *R. roxburghii* genotypes by widely targeted metabolomics and showed that flavonoids, triterpenoids, and phenolic acids have an antioxidant capacity [6]. The flavonoids in *R. roxburghii* fruit also exhibit critical biological activities, such as anti-inflammation [7], the expansion of coronary arteries, the control of blood pressure, and the protection of blood vessels [8]. Polyphenols from *R. roxburghii* ameliorate the symptoms of diabetes by activating insulin [9]. Because of its nutritional and medical functions, the economic cultivation area of *R. roxburghii* has expanded to 140,000 ha in the Guizhou province. Several enterprises have participated in processing *R. roxburghii*, and >50 products have been explored.

The physiological and biochemical studies of *R. roxburghii* fruits are rather intensive; however, the monomers at the developmental stage of *R. roxburghii* and the molecular mechanism of the accumulation have not been analyzed or reported. Transcriptome is an effective method to obtain almost all transcripts of *R. roxburghii* fruits in a specific state. Transcriptome analysis is widely used in *R. roxburghii* [10,11,12,13,14]. Among the biochemical substances, only AsA [10,13] and lignin [11] have been investigated by transcriptome analysis. The studies used the transcriptome to identify the key structural genes and transcription factors to reveal their accumulation. The synthesis of other crucial metabolites, such as amino acids, phenolic acids, and flavonols, is yet to be studied.

In recent years, metabolomics integrated with transcriptomics has been widely used to elucidate the biosynthesis pathways of metabolites in plants [15,16]. Hitherto, this technology has been widely applied to analyze the key genes in watermelon [17], pear [18], and grape [19]. The present study combined metabolomics and transcriptomics to elucidate the amino acids, phenolic acids, and flavonol derivative biosynthesis network and their antioxidation activities during *R. roxburghii* fruit development. Furthermore, the key transcription factors were identified, and the metabolites contributing to the in vitro antioxidative activities were screened. Taken together, these approaches provide a novel insight into the identification of the key metabolites and their regulation in *R. roxburghii* fruits.

## 2. Materials and Methods

### 2.1. Plant Materials

The ‘Guinong 5’ plants were 7 years old and grown in the outdoor fruit germplasm repository of Guizhou University, Guizhou, China (26°42.408′ N, 106°67.353′ E). The fruits were harvested in 2017 at three different developmental stages: 30, 60, and 90 days after anthesis (DAA) (Figure S1), which represented the young fruit stage, development stage, and mature stage noted as Rr5-30, Rr5-60, and Rr5-90, respectively. Three biological replicates were sampled for each stage, resulting in a total of nine samples. Each sample had 20–60 fruits. Moreover, the mature fruits of the other five genotypes, Rr-1, Rr-3, Rr-4, Rr-7, and Rr-f, were collected as described by Jiang et al. [6]. The samples were frozen in liquid nitrogen and stored at −80 °C until use.

### 2.2. Determination of Bioactive Substance Content and Antioxidant Capacity

The total phenol content was determined using the Folin–Ciocalteau reagent [20]. The flavonoid content was analyzed using the spectrophotometric method, as described by Huang et al. [21]. The AsA was determined according to the method of An et al. [1]. Triterpenoids were determined by vanillin–glacial acetic acid colorimetry [22]. The levels of organic acids and lipids were measured as described in the *Principles and Techniques of Plant Physiological Biochemical Experiment* [23]. The amino acid content was determined using Suzhou Greis Biotechnology Co., Ltd (Suzhou, China). kits. In order to determine the DPPH (1,1-Diphenyl-2-picrylhydrazyl Free Radical) radical-scavenging activity, the protocol by Andrés et al. [24] was employed with minor modifications. Martinez et al.’s [25] method was used to determine ABTS (2,2’-Azinobis-3-ethylbenzthiazoline-6-sulphonate) cation radical-scavenging activity, and Benzie and Strain’s method [26] was applied to determine the ferric ion-reducing activity. The antioxidant activity was expressed in μmol Trolox equivalents (TE)/g fresh mass.

### 2.3. Metabolite Extraction and ESI-Q TRAP-MS/MS Analysis

The frozen fruits were crushed with a churn (1.5 min, 30 Hz, three repetitions, 400 mm, rage). Then, an equivalent to 100 mg powder samples was extracted using a 70% methanol solution containing 0.1 mg of lidocaine at 4 °C overnight. The supernatant obtained by centrifugation of the extract at 10,000× *g*, 4 °C for 10 min, was filtered through a 0.2286-micron hydrophilic Teflon syringe filter (SCAA-104, Amperale, Shanghai, China) and subjected to metabolomics analysis. The quality control samples were injected every two samples (mixtures 1–3) to obtain a dataset, and the repeatability was evaluated.

An LC-ESI-MS/MS system (HPLC, UFLC CBM30A system; mass spectrometry, Applied Biological System 4500 Q TRAP, AB Sciex, Foster City, CA, USA) and Agilent 6520 accurate mass time-of-flight mass spectrometry (AB Sciex, Foster City, CA, USA) were used to evaluate the chromatographic separation by mobile phase A (deionized water 0.04% acetic acid) and mobile phase B (acetonitrile 0.04% acetic acid × acetonitrile 0.04% acetic acid) on an ACQUITY UPLCHSST3C 18 column (2.1 mm × 100 1.8 μm; Waters, MA, USA).

The mass data were acquired in the electrospray ionization-positive/negative mode using the following parameters: ion spray voltage 5.5 kV; 55 pounds/square inch ion-derived gas iodine (GSI); 60 pounds/square inch gas II (GSII); 25 pounds/square inch curtain gas; 550 °C temperature of the turbine spray; instrument tuning and mass calibration with 10 and 100 μmol/L polypropylene glycol solution in triple quadrupole (QQQ) and linear ion trap (LIT) modes, respectively. The specific diffusion potential and collision energy optimization were carried out to assess the diffusion potential and collision energy of a single multi-reaction monitoring transition.

### 2.4. Qualitative and Quantitative Analysis of Metabolites

Based on the self-built metadata database (MWDB), material characterization was carried out according to the secondary spectral information. Metabolite quantification is a multi-response monitoring model (multiple reaction monitoring) that uses the mass spectrometry data of triple and four-stage rods multiple reaction monitoring (MRM). Based on fold change ≥2 (upregulated) or ≤0.5 (downregulated) and *p*-value < 0.05, the significantly changed metabolites (SCMs) were screened. SCM principal component analysis was performed on platform (www.r-project.org, accessed on 11 May 2020) to study the variety-specific accumulation of metabolites. Then, these differential metabolites were screened using a threshold variable importance in projection (VIP) value (VIP ≥ 0.8) from the orthogonal partial least squares discriminant analysis (OPLS-DA) model. The annotated metabolites were mapped to the KEGG pathway database (http://www.kegg.jp/kegg/pathway.html, accessed on 13 May 2020) to determine the pathway associations. Pathway enrichment analysis was performed on the web-based server Metabolite Sets Enrichment Analysis (MSEA; http://www.msea.ca, accessed on 15 May 2020). The pathways with Bonferroni-corrected *p*-values ≤ 0.05 were considered significantly enriched.

### 2.5. Transcriptome Information and Prediction of Transcription Factors

The data on the lignin of *R. roxburghii* were obtained from Lu et al. [11]. The transcriptome of the three samples was sequenced at 30, 60, and 90 days after flowering. The fruits were harvested in 2017, and denoted as 30 DAA, 60 DAA, and 90 DAA, respectively. The transcription factors were identified by the predicted peptide sequences of all transcripts searched against the transcription factors in PlantTFDB 3.0 using the Transcription Factor Prediction module (http://planttfdb.cbi.pku.edu.cn/prediction.php, accessed on 14 May 2020) with default parameters. Then, the promoter elements were predicted using the Plantcare website (http://bioinformatics.psb.ugent.be/webtools/plantcare/html, accessed on 8 February 2021).

### 2.6. Quantitative RT-PCR Analysis

Total RNA was extracted from the fruit samples using TRIzol reagent (Invitrogen, Shanghai, China), according to the manufacturer’s instructions. The primer sequences are listed in Table S6. qRT-PCR was performed on an ABI ViiA 7 DX system (Thermo Fisher Scientific, MA, USA) using SYBR Premix Ex Taq II (TaKaRa, Dalian, China) with the *ubiquitin* gene as an endogenous control. The data were analyzed using the 2^−ΔΔCT^ method [27]. The mean expression and standard deviation (SD) were calculated based on the results of three independent experiments.

### 2.7. Statistical Analysis

The data were analyzed statistically with SPSS 25.0 (SPSS Inc., Chicago, IL, USA). Analysis of variance was used to test any difference in bioactive substance content and antioxidant activities resulting from these methods. Duncan’s new multiple range test was used to determine significant differences. Correlations among the data obtained were calculated using Pearson’s correlation coefficient (r). Heatmap and principal components analysis (PCA) were made using the pheatmap and factoextra packages, respectively, within R v3.5.2 (R Core Team. R: A Language and Environment for Statistical Computing, R Foundation for Statistical Computing, Vienna, Austria, 2015).

## 3. Results

### 3.1. Bioactive Substance Content and Antioxidant Capacity in R. roxburghii during Fruit Development

**Figure 1:**
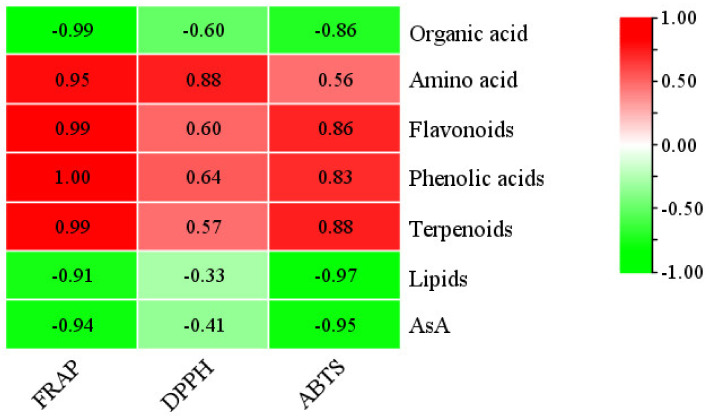
Correlation coefficient between active substances and antioxidant capacity. High correlation corresponds to red, and low correlation corresponds to green. When the correlation coefficient is ≥0.95, it is significant at the level of 0.05; when the correlation coefficient is ≥0.99, it is significant at the level of 0.01.

The content of organic acids, amino acids, flavonoids, total phenols, terpenoids, AsA, lipids, DPPH radical-scavenging activity (DPPH), ABTS cation radical-scavenging activity (ABTS), and the ferric ion-reducing activity (FRAP) were measured at 30 (Rr5-30), 60 (Rr5-60), and 90 (Rr5-90) DAA of *R. roxburghii* fruits (Table 1). The content of flavonoids, total phenols, terpenoids, and FRAP showed a similar trend, i.e., decreased significantly with fruit development. Correlation analysis verified that the FRAP was the most positively correlated with total phenols, flavonoids, and terpenoids (*p* < 0.01) (Figure 1). Similarly, the content of amino acids decreased with fruit development. The subtle difference was that there was no significant variation between Rr5-60 and Rr5-90 (Table 1). Hence, the content of amino acids also showed a significant positive correlation with the FRAP value (*p* < 0.05) (Figure 1). Taken together, the content of amino acids, flavonoids, total phenols, and terpenoids was positively correlated with three in vitro antioxidative activities during *R. roxburghii* fruit development (Figure 1).

### 3.2. Metabolome Profiling of R. roxburghii Fruits at Different Ripening Stages

In order to better understand the metabolites in the fruit development stage of *R. roxburghii* fruit, we performed a widely targeted LC-MS/MS-based metabolite profiling of Rr5-30, Rr5-60, and Rr5-90, respectively. Subsequently, principal component analysis (Figure S2A) and sample correlation analysis (Figure S2B) were carried out to evaluate the repeatability of the data. The principal component analysis showed that in the PC1×PC2 score chart, three samples related to the quality control (QC) samples were clearly separated, indicating differences in the metabolomes between the sample groups [24], while the sample correlation analysis showed high correlation among biological replicates and low correlation between different biological replicates, suggesting satisfactory data.

**Figure 2:**
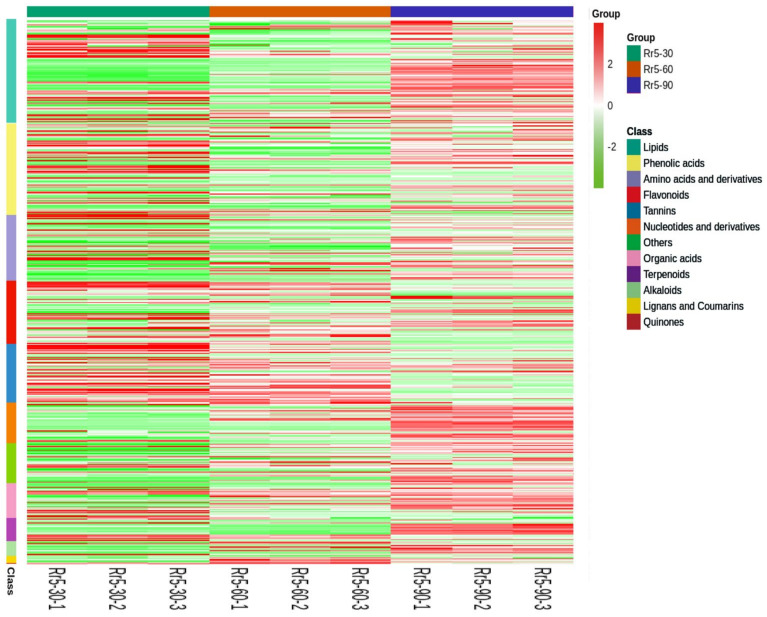
Heatmap of all detected metabolites. Horizontal: sample name, longitudinal: metabolite classification situation. Different colors represent the values for standardized treatments of relative content, ranging from low (green) to high (red).

A total of 533 metabolites were identified and clarified in 12 types, including lipids (102), amino acids and derivatives (64), flavonoids (62), tannins (57), nucleotides and derivatives (40), phenolic acids (90), organic acids (34), terpenoids (23), alkaloids (14), lignans and coumarins (7), quinones (1), and others (39) (Table S1). After the unit variance scale (uv) normalization of 533 metabolites’ data, we drew the heatmap using the R software (Figure 2), which showed that the variation tendency of the monomer content in each of the 12 types was not consistent during *R. roxburghii* fruit development, while most of the flavonoids and tannins were decreased.

### 3.3. Differentially Accumulated Metabolites between Young and Mature Fruits of R. roxburghii

**Figure 3:**
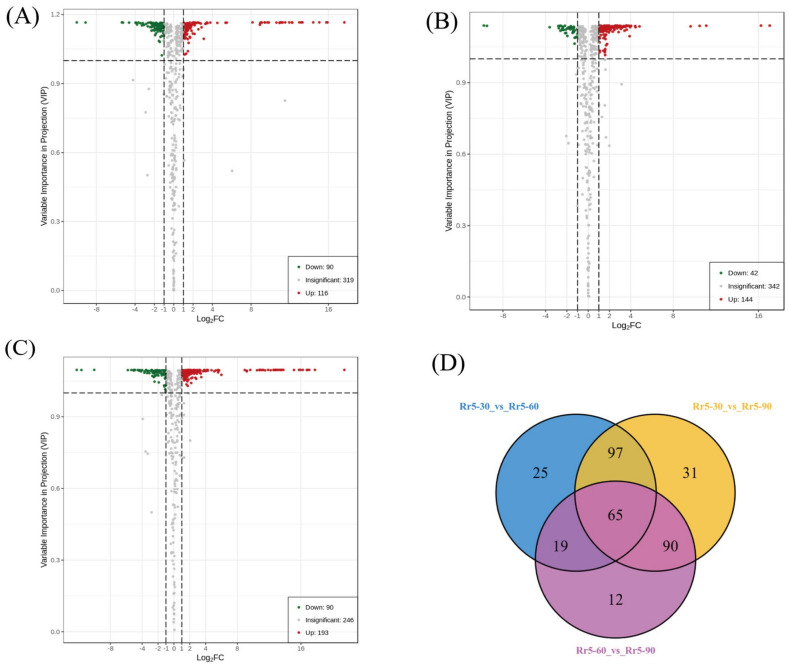
Differential accumulation of metabolites. Differential metabolites were defined as metabolites with fold-change ≥2 or ≤0.5 in the comparison of two samples and fulfilling the condition of VIP≥1. (**A**) Rr5-30 vs. Rr5-60 volcano plot. (**B**) Rr5-60 vs. Rr5-90 volcano plot. (**C**) Rr5-30 vs. Rr5-90 volcano plot. (**D**) Venn diagram. Each circle represents a comparison group, with the numbers in the overlapping part of the circle; the circle represents the number of SCMs shared between the comparison groups. The number without overlap represents the number of unique SCMs in the comparison group.

To identify the SCMs among Rr5-30, Rr5-60, and Rr5-90, we selected fold-change ≥2 (upregulated) or ≤0.5 (downregulated) metabolites. These metabolites were screened using VIP ≥ 1 from the OPLS-DA model. A total of 339 substances changed during the *R. roxburghii* fruit development (Figure 3D). Then, 206 SCMs were identified between the fruits of Rr5-30 and Rr5-60 (Figure 3A), of which 90 were downregulated, and 116 were upregulated. Furthermore, 186 SCMs were identified between the Rr5-60 and Rr5-90 fruits (Figure 3B), including 42 downregulated and 144 upregulated SCMs. Between the fruits of Rr5-30 and Rr5-90, 283 SCMs were identified (Figure 3C), of which 90 were downregulated and 193 were upregulated. Finally, 65 metabolites changed significantly in the three compared groups (Figure 3D).

Next, we mapped the SCMs to the KEGG database. The results showed the metabolic pathways were involved with the highest number of metabolites between Rr5-30 and Rr5-60 (Figure S3A) or between Rr5-60 and Rr5-90 (Figure S3B) and between Rr5-30 and Rr5-90 (Figure S3C), followed by the biosynthesis of secondary metabolites and amino acids.

**Figure 4:**
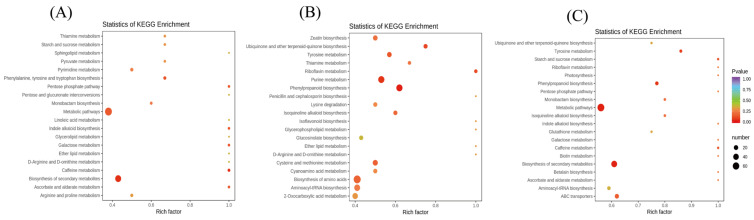
KEGG enrichment analysis of differentially accumulating metabolites. (**A**) Differentially accumulating metabolites between Rr5-30 and Rr5-60. (**B**) Differentially accumulating metabolites between Rr5-60 and Rr5-90. (**C**) Differentially accumulating metabolites between Rr5-30 and Rr5-90. Each circle in the plot represents the number of associated metabolites and is the position according to its abundance factor. The *p*-values are indicated by colors.

Subsequently, we performed a KEGG pathway enrichment analysis to identify the differences in the metabolic pathways between the three groups. The enrichment analysis suggested that the metabolic pathways and biosynthesis of secondary metabolites differed significantly in the three groups (*p* < 0.05) (Figure 4). Notably, significant differences were observed in the biosynthesis of amino acids, purine metabolism, and phenylpropanoid biosynthesis between Rr5-60 and Rr5-90 (Figure 4B).

The characteristic metabolites were the intersections of different groups of various metabolites. At this stage, the characteristic metabolites need to meet the condition that the content was more than at other stages. A total of 59 characteristic metabolites were identified in Rr5-30, 6 in Rr5-60, and 120 in Rr5-90 (Table S2). These results suggested that the metabolites differed significantly between Rr5-30 and Rr5-90, while Rr5-60 may be at the middle stage of maturity. The characteristic metabolites were involved in phenolic acids, flavonoids, amino acids and derivatives, organic acids, nucleotides and derivatives, tannins, and lipids. Importantly, flavonoids and amino acids, such as dihydroquercetin (taxifolin), naringenin chalcone, L-serine (Ser), L-asparagine (Asn), L-(−)-threonine (Thr), 2,6-diaminoopimelic acid (2,4-DAP), and L-citrulline (Cit), need to be investigated in depth in Rr5-30. For Rr5-90, phenolic acids and amino acids, such as chlorogenic acid, cinnamic acid, p-coumaric acid, caffeic acid, ferulic acid, γ-aminobutyric acid (GABA), L-(+)-arginine (Arg), 5-aminovaleric acid (5-AVA), L-homocysteine (Hcy), L-methionine (Met), S-(5′-adenosy)-L-homocysteine (SAH), L-(+)-lysine (Lys), L-valine (Val), L-histidine (His), L-(−)-tyrosine (Tyr), and L-tyramine (TA), are prominent.

### 3.4. Conjoint Analysis of Transcriptome and Metabolome

**Figure 5:**
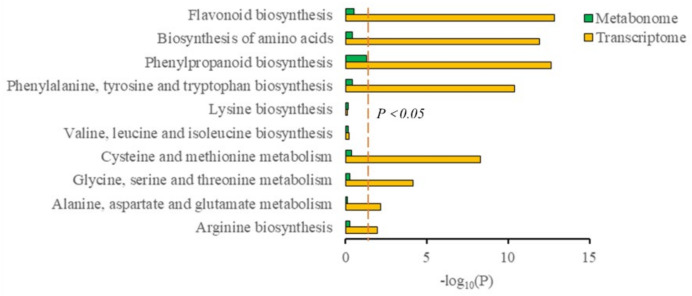
Histogram of the KEGG pathway enriched by the differentially expressed genes (DEGs) and metabolites between the two groups. Each post represents a pathway: orange shows the transcriptome, and green represents the metabolome. The ordinate represents pathway enrichment, and the abscissa represents the target pathway. The parameter “*p*-value < 0.05” was used as the threshold to judge the significance of gene expression and the difference in the metabolites.

The conjoint analysis of the transcriptome and metabolome was combined with the transcriptome data [11] to screen out the essential genes and metabolites. The KEGG enrichment analysis showed the co-enrichment pathways of DEGs and SCMs through conducted *p*-values (Figure 5). The co-enrichment is involved in nine pathways, including the biosynthesis of amino acids, arginine biosynthesis, alanine, aspartate, and glutamate metabolism, glycine, serine, and threonine metabolism, cysteine and methionine metabolism, and the biosynthesis of valine, leucine, isoleucine, lysine, phenylalanine, tyrosine, tryptophan, phenylpropanoid, and flavonoids.

### 3.5. Biosynthesis of Amino Acids, Phenolic Acids, and Flavonol Derivatives

**Figure 6:**
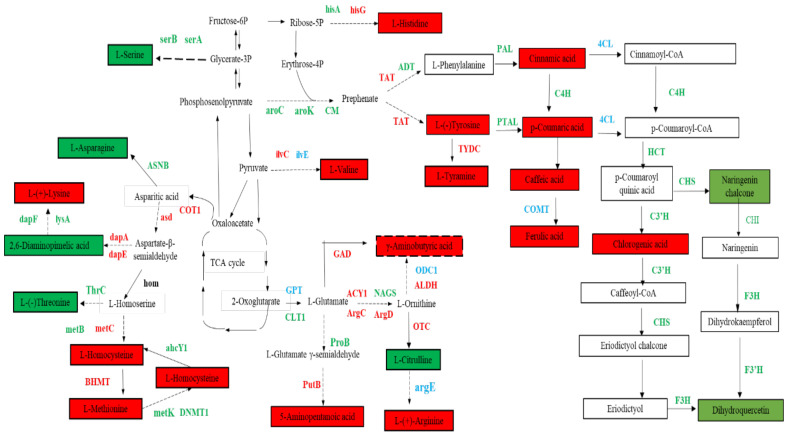
Changes in the characteristic metabolites and DEGs in the synthesis pathway of amino acids, phenolic acids, and flavonol derivatives. Color indicates the change in related substances from 30 DAA to 90 DAA; red indicates increased, green indicates decreased, and blue indicates both increased and decreased.

Differential expression analysis was performed using the DESeq R package (1.10.1). The resulting *p* values were adjusted using Benjamini and Hochberg’s approach for controlling the false discovery rates. Genes with an adjusted *p*-value < 0.05 identified using DESeq were considered as differentially expressed genes (DEGs). We identified 48 enzymes and 74 DEGs, including 10 structural genes involved in the phenylpropanoid biosynthesis, 52 in the biosynthesis of amino acids, and 12 in flavonoid biosynthesis in the transcriptome (Figure 6, Table S3).

Moreover, two potential key structural genes were identified in the phenylpropanoid biosynthesis pathway. The gene (*evm.model.Contig428.373*) was strongly correlated with cinnamic acid, p-coumaric acid, caffeic acid, and ferulic acid (R^2^ = 0.888; R^2^ = 0.851; R^2^ = 0.959; R^2^ = 0.886) (Table S4), which was identified as 4-coumarate-CoA ligase (*4CL*). *4CL* (*evm.model.Contig428.373)* was the upstream gene of these metabolites. The results showed that the high expression of the *4CL* (*evm.model.Contig428.373*) gene might result in a high accumulation of these metabolites. In addition, a significant positive correlation was established between ferulic acid and *evm.model.Contig179.433* (R^2^ = 0.922), identified as catechol O-methyltransferase (*COMT*), indicating a critical effect of *COMT* (*evm.model.Contig179.433*) on ferulic acid synthesis.

Next, 12 potential key structural genes were identified in the pathway of flavonoid biosynthesis. Four genes (*evm.model.Contig324.104*; *evm.model.Contig324.107*; *evm.model.Contig324.105*; *evm.model.Contig110.47*) were identified as naringenin–chalcone synthase (*CHS*) and two genes (*evm.model.Contig4.33*, *evm.model.Contig280.68*) were identified as shikimate O-hydroxycinnamoyltransferase (*HCT*), the four *CHS* and two *HCT* positively effectuated on naringenin chalcone and dihydroquercetin. Moreover, the four *CHS*, two *HCT*, one coumaroylquinate 3’-monooxygenase gene (*C3’H*; *evm.model.Contig374.36*), two chalcone isomerase genes (*CHI*; *evm.model.Contig317.48*, *evm.model.Contig319.126*), one flavanone 3-hydroxylase gene (*F3H*; *evm.model.Contig281.218*), and one flavonoid 3’-monooxygenase gene (*F3’H*; *evm.model.Contig418.464*) exert a positive effect on chlorogenic acid (Table S4). The results showed that these genes were crucial for the accumulation of naringenin chalcone, dihydroquercetin, and chlorogenic acid, but chlorogenic acid was negatively correlated with *F3’H* (*evm.model.Contig401.234*).

Furthermore, 18 potential key structural genes were screened in the pathway of the biosynthesis of amino acids (Table S4). The branched-chain amino acid aminotransferase gene (*ilvE*; *evm.model.Contig352.46*) had a positive correlation with Val, while Ser was positively correlated with phosphoserine phosphatase genes (*serB*; *evm.model.Contig418.450*). The high expression of ATP phosphoribosyltransferase (*hisG*; *evm.model.Contig229.63*) has a positive impact on the high accumulation of His. tyrosine aminotransferase gene (*TAT; evm.model.Contig389.26*), which was associated with Tyr (R^2^ = 0.832) and TA (R^2^ = 0.815), indicating that the accumulation of Tyr and TA was affected by *TAT* (*evm.model.Contig389.26*). The glucose-6-phosphate/phosphate translocator (*GPT; evm.model.Contig339.70*), acetylornithine aminotransferase gene (*ACY1*; *evm.model.Contig290.142*), and aldehyde dehydrogenase gene (*ALDH*; *evm.model.Contig68.59*) were found, and thus positively correlated with GABA. *GPT* (*evm.model.Contig339.70*) positively influenced the accumulation of Arg and 5-aminopentanoic acid. *ACY1* (*evm.model.Contig290.142*) and *argG* (*evm.model.Contig403.2*) positively affected the accumulation of Arg. Cit was positively affected by glutamate synthase (*GLT1; evm.model.Contig386.230*) and amino-acid acetyltransferase (*NAGS*; *evm.model.Contig273.43)*. Notably, *argG* (*evm.model.Contig403.2*) was detected in the synthesis pathway from Cit to Arg, wherein Cit decreased while Arg increased. Therefore, we speculated that the low expression of *argG* (*evm.model.Contig403.2*) increases the activity of argG. Interestingly, *COT1* (*evm.model.Contig120.160; evm.model.Contig283.202*) and aspartate–semialdehyde dehydrogenase (*asd; evm.model.Contig332.117*) were upstream genes in the synthesis of aspartic acid family amino acids that affected the accumulation of many metabolites, such as Hcy, Met, SAH, Thr, 2,4-DAP, and Lys. Moreover, the accumulation of Hcy was also positively affected by *metC* (*evm.model.Contig243.89*). Met and SAH were also positively affected by cystathionine beta-lyase (*metC; evm.model.Contig243.89*) and homocysteine S-methyltransferase genes (*BHMT; evm.model.Contig254.103*, *evm.model.Contig40.474*). The asparagine synthetase gene (*ASNB;* evm.model.Contig247.176) might be a critical step for Asn synthesis. We also observed that diaminopimelate decarboxylase (*lysA; evm.model.Contig284.69*) was positively correlated to 2,4-DAP and linked to catabolism. Therefore, we speculated that *lysA* reduces lysA activity, accelerates the metabolic process, and reduces the content of 2,4-DAP. Thr was affected by the synthesis pathway, indicating a positive correlation with threonine synthase gene (*thrC; evm.model.Contig264.74*). In addition, Thr was affected by the competition of other pathways and, hence, was negatively correlated with *metC* (*evm.model.Contig243.89*).

In order to further verify the reliability of the data, we randomly selected 15 genes involved in the amino acid, phenylpropanoid, and flavonoid biosynthesis. The qRT-PCR results showed that the expression of 15 genes was consistent with the RNA-seq data (Figure S4).

### 3.6. RrMYBs in the Regulation of Amino Acid, Phenolic Acid, and Flavonol Derivative Biosynthesis

**Figure 7:**
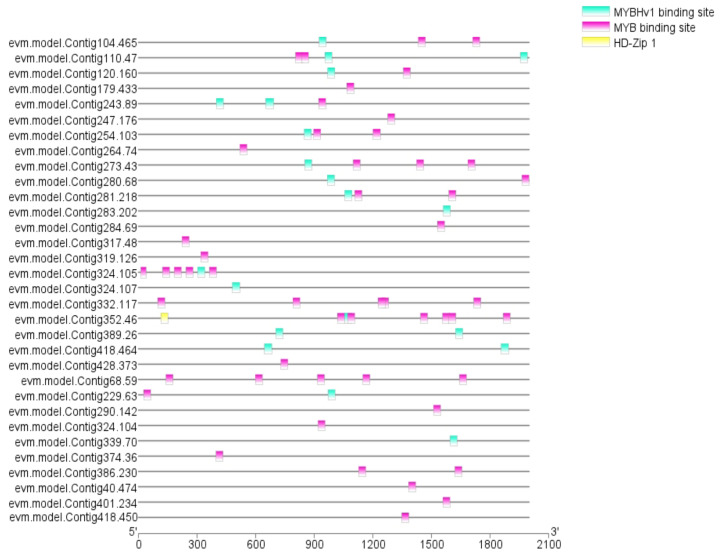
Promoter element analysis of 32 potential structural genes.

In order to explore the mechanism that might affect the amino acids, phenolic acids, and flavonoids in *R. roxburghii* fruit, we analyzed the promoter elements of the above 32 potentially critical structural genes to collect information about the transcription factors. The results showed that the MYB family regulated the biosynthesis of amino acids, phenolic acids, and flavonoid derivatives of *R. roxburghii* fruits. In addition, Val may be regulated by HD-Zip transcription factors (Figure 7).

**Figure 8:**
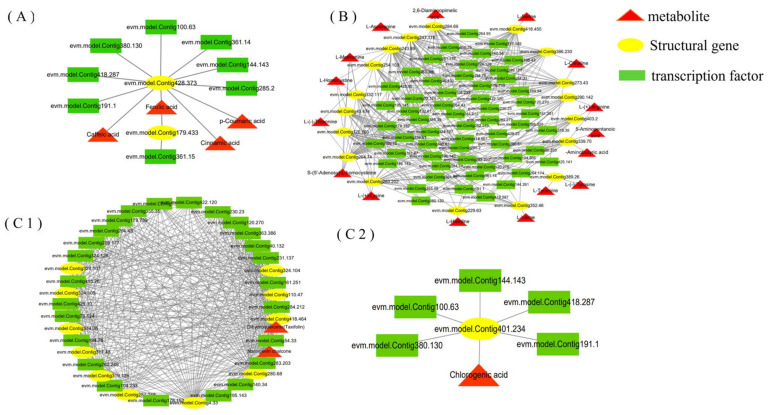
Regulatory network of amino acid, phenolic acid, and flavonol derivative biosynthesis. (**A**) The regulatory correlation between the characteristic metabolites of the phenolic acid pathway and structural genes and transcription factors. (**B**) The regulatory correlation of the amino acid pathway. (**C1**) The regulatory correlation of the flavonoid pathway (naringenin chalcone and dihydroquercetin). (**C2**) The regulatory correlation of the flavonoid pathway (chlorogenic acid).

Based on the transcriptome, we identified 158 MYB transcription factors, of which 75 were DEGs. According to the correlation analysis, 48 DGEs were potentially involved in the regulation of the characteristic metabolites of *R. roxburghii* fruits. Furthermore, we analyzed the interaction network among the 32 structural genes, 48 *RrMYBs*, and 23 metabolites using the Cytoscape software, Institute for Systems Biology, Seattle, WA, USA (Figure 8).

The network results showed that eight *RrMYBs* are involved in the regulation of phenolic acids (Figure 8A), of which seven *RrMYBs*, such as *evm.model.Contig 380.130, evm.model.Contig 191.1*, and *evm.model.Contig 361.14*, acting on the structural gene (*evm.model.Contig 428.373*), regulated cinnamic acid, p-coumaric acid, caffeic acid, and ferulic acid. In addition, ferulic acid was also regulated by 1 *RrMYB* (*evm.model.Contig 361.15*). Subsequently, 30 *RrMYBs* were found to be potentially involved in the flavonoid synthesis pathway (Figure 8C), and 5 *RrMYBs* regulated the synthesis of chlorogenic acid acting on the structural gene (*evm.model.Contig 428.373*), while dihydroquercetin (taxifolin) and naringenin chalcone were regulated by many *RrMYBs*, such as *evm.model.Contig415.76*, *evm.model.Contig40.132*, and *evm.model.Contig422.120* (Table S5). Amino acids and their derivatives were regulated by 54 DGEs, and the network showed a complex correlation (Figure 8B). Multiple RrMYBs regulated one metabolite, and one *RrMYB* regulated multiple structural genes and metabolites simultaneously. For example, *evm.model.Contig418.287* regulated several metabolites, such as GABA, Arg, 5-AVA, Hcy, Met, SAH, Lys, His, and Val. Arg was also regulated by many *RrMYBs*, such as *evm.model.Contig319.35*, *evm.model.Contig80.203*, *evm.model.Contig418.287*, *evm.model.Contig284.79*, *evm.model.Contig144.143*, *evm.model.Contig264.174*, *evm.model.Contig100.63*, *evm.model.Contig380.130*, *evm.model.Contig361.15*, and *evm.model.Contig191.1*.

Although there are many *RrMYBs* involved in the regulation of substances, our analysis found that six *RrMYBs* regulate 9–15 substances (Table S6) that are the potential key transcription factors, including *evm.model.Contig418.287* (*RrMYB62*), *evm.model.Contig144.143* (*RrRAX3*), *evm.model.Contig100.63* (*RrREVEILLE7*), *evm.model.Contig361.14* (*RrMYB105*), *evm.model.Contig380.130* (*RrMYB108*), and *evm.model.Contig191.1* (*RrLHY*).

### 3.7. Key RrMYBs Expression and Metabolites in Different R. roxburghii Genotypes

**Figure 9:**
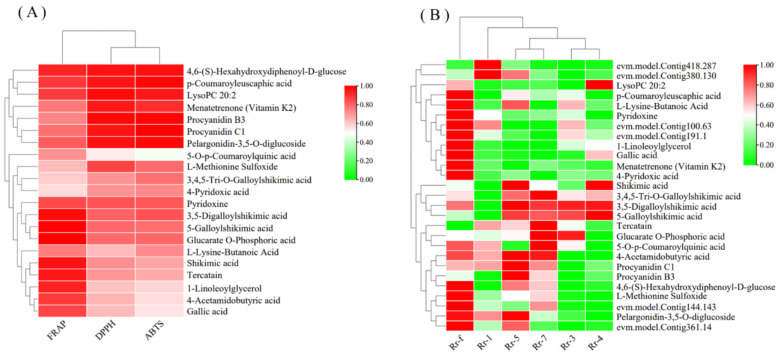
Heatmap of relevant indicators. (**A**) Correlation coefficient between key metabolites and antioxidant capacity. High correlation corresponds to red, and low correlation corresponds to green. When the correlation coefficient is ≥0.7, it is significant at the level of 0.05; when the correlation coefficient ≥0.8, it is significant at the level of 0.01. (**B**) Heatmap of the key metabolites and *RrMYBs*. Different colors represent the values for standardized treatments of relative content, ranging from low (green) to high (red).

Combined with the metabolomics data of different *R. roxburghii* genotypes reported by Jiang et al. [6], 360 metabolites were detected in the current and Jiang et al.’s studies [6]; among these, 21 metabolites are significantly positively correlated with the in vitro antioxidative activities (Figure 9A). Of the 21 metabolites, five were phenolic acids (4,6-(S)-hexahydroxydiphenoyl-D-glucose, 3,4,5-tri-O-galloylshikimic acid, 3,5-digalloylshikimic acid, 5-galloylshikimic acid, and 5-O-p-coumaroylquinic acid), four were tannins (procyanidin C1, procyanidin B3, gallic acid, and tercatain), one was a flavonoid (pelargonidin-3,5-O-diglucoside), two were amino acids and derivatives (Met sulfoxide (MetO) and L-lysine-butanoic acid), two were lipids (lysoPC 20:2 and 1-linoleoylglycerol), two were organic acids (4-acetamidobutyric acid and shikimic acid), one was a terpenoid (p-coumaroyleuscaphic acid), and four were others (4-pyridoxic acid, pyridoxine, menatetrenone (vitamin K2), and glucarate O-phosphoric acid). These were considered key metabolites that contributed to the in vitro antioxidative activities in the fruits at various development stages or in the fruits of different *R. roxburghii* genotypes.

Subsequently, six *RrMYBs* were selected as potential key regulatory factors from the network that related to the amino acids, phenolic acids, and flavonol derivatives during *R. roxburghii* fruit development (Figure 8; Table S6). The relative expression of these molecules was detected in the fruits of six *R. roxburghii* genotypes. After data standardization, the trend clustering was carried out through the heatmap consisting of 21 key metabolites and six key *RrMYBs* in the fruits of six *R. roxburghii* genotypes (Figure 9B). Among these, the expression of four *RrMYBs* showed a similar variation trend, with some key metabolites, such as *evm.model.Contig361.14* (*RrMYB105*) and the pelargonidin-3,5-O-diglucoside, *evm.model.Contig144.143* (*RrRAX3*) and MetO, *evm.model.Contig191.1* (*RrLHY*), *evm.model.Contig100.63* (*RrREVEILLE7*), and pyridoxine, indicating that these *RrMYBs* significantly affect various metabolites in the network during fruit development (Figure 8) and the key metabolites related to the in vitro antioxidation activities in the fruits of six *R. roxburghii* genotypes (Figure 9B).

## 4. Discussion

In the present study, organic acids, amino acids, flavonoids, total phenols, terpenoids, AsA, and lipids were measured at 30 (Rr5-30), 60 (Rr5-60), and 90 (Rr5-90) days after anthesis (Table 1). Significant variation was observed at different developmental stages except for lipids. Organic acids increased from 0.58% at Rr5-30 to 1.12% at Rr5-90, AsA increased from 70.78 mg/100g at Rr5-30 to 861.13 mg/100g at Rr5-90, which was similar to the results found by An et al. [1]. Amino acids were the highest at Rr5-30, but there was no significant decrease between Rr5-60 and Rr5-90. Flavonoids, phenolic acids, and terpenoids decreased sharply in terms of fruit growth. Previous studies also showed the same results [5]. Meanwhile, we detected DPPH, ABTS, and FRAP (Table 1). The results showed that the changes in the three antioxidant activities were different in the *R. roxburghii* growth, but these activities were the highest in Rr5-30. Correlation analysis verified that the FRAP was most positively correlated with total phenols, flavonoids, and terpenoids (*p* < 0.01) (Figure 1). The results of the computing correlations presented in Figure 1 are in accordance with previous studies on *R. roxburghii* fruits [5]. The content of total phenols and flavonoids was 1701 mg/100 g and 456 mg/100 g, respectively, in mature fruit, and the DPPH, FRAP, and ABTS antioxidant activity was 66.04 mmol TE/g, 141 mmol TE/g, and 66 mmol TE/g (Table 1), respectively. The results showed that the values of total phenol, flavonoids, DPPH, FRAP, and ABTS in *R. roxburghii* fruit were higher than common fruits rich in these bioactive substances, such as citrus [28], grape [29,30], and *Lonicera caerulea* [31].

We identified 533 metabolites during the development of the *R. roxburghii* “Rr-5” fruit, which was fewer than the metabolites in the six different *R. roxburghii* genotypes identified by Jiang et al. [6], indicating that different genotypes of *R. roxburghii* exhibited significant differences in metabolites. Guo et al. detected 134 metabolites grouped into 11 classes in blueberry [32]. Chen et al. identified 407 metabolites in strawberry [33], while 352 metabolites were detected and identified in citrus [34]. Ma et al. identified 464 metabolites in berry skin [35]. Thus, *R*. *roxburghii* fruit is rich in secondary metabolites, and we can often consume the products in our daily life, such as dried *R*. *roxburghii* fruit, *R*. *roxburghii* juice, and *R*. *roxburghii* wine. Furthermore, we combined their data to confirm the in vitro antioxidant activity-related metabolites in both the developing *R. roxburghii* “Rr-5” fruits and six fruits of a different genotype. Consistent with the previous studies [5,6], total phenols, flavonoids, and triterpenoid were suggested as the key metabolites, contributing to three in vitro antioxidant activities (FRAP, DPPH, and ABTS). Total phenols included phenolic acids, flavonoids, and tannins. Thus, tannins should also be considered, which were not mentioned by Jiang et al. [6]. These phenol metabolites did not present a consistent trend (Figure 2). A total of five phenolic acids (4,6-(S)-hexahydroxydiphenoyl-D-glucose, 3,4,5-tri-O-galloylshikimic acid, 3,5-digalloylshikimic acid, 5-galloylshikimic acid, and 5-O-p-coumaroylquinic acid), four tannins (procyanidin C1, procyanidin B3, gallic acid, and tercatain), one flavonoid (pelargonidin-3,5-O-diglucoside), and one terpenoid (p-coumaroyleuscaphic acid) were selected as key metabolites in both the developing *R. roxburghii* “Rr-5” fruits and six fruits of a different genotype (Figure 9A). To the best of our knowledge, 4,6-(S)-hexahydroxydiphenoyl-D-glucose and p-coumaroyleuscaphic acid were reported for the first time as the critical antioxidant substances. Moreover, 3,4,5-tri-O-galloylshikimic acid, 3,5-digalloylshikimic acid, and 5-galloylshikimic acid were evaluated as potential inhibitors of human immunodeficiency virus (HIV) replication and anti-influenza agents [36,37], and 5-O-p-coumaroylquinic acid and gallic acid protect HaCaT human keratinocytes from UVB-induced damage [38]. A previous study has shown the neuroprotective effect of procyanidin C1 and procyanidin B3 [39]. Tercatain acts as a potential anti-viral component [40]. Li et al. found that pelargonidin-3,5-o-diglucoside improves the antioxidant activity of cells by analyzing the thermal stability of the substances [41]. In this study, *evm.model.Contig361.14* (*RrMYB105*) and the pelargonidin-3,5-O-diglucoside clustered into one category owing to their similar trend (Figure 9B), indicating that the accumulation of pelargonidin-3,5-O-diglucoside is regulated directly by *evm.model.Contig361.14* (*RrMYB105*) in *R. roxburghii* fruits.

Moreover, two lipids (lysoPC 20:2 and 1-linoleoylglycerol), two organic acids (4-acetamidobutyric acid and shikimic acid), and four others (4-pyridoxic acid, pyridoxine, menatetrenone (vitamin K2), and glucarate O-phosphoric acid) were selected as key metabolites in both the developing *R. roxburghii* “Rr-5” fruits and six other fruits of different genotypes (Figure 9A). These were not consistent with the analysis of physiological data (Figure 1), reflecting the differences between the whole and the individual (Table 1; Figure 2). A recent study showed that lysoPC 20:2 relieves the symptoms of gastrointestinal disorder by increasing the bacteria Ruminococcaceae and decreasing Streptococcaceae, Erysipelotrichaceae, and Lachnospiraceae in the fecal microbiota [42]. In addition, 1-linoleoylglycerol showed a high level of antioxidant activity in *Morchella esculenta* Pers [43], while 4-acetamidobutyric acid was involved in chronic fatigue syndrome [44] and pediatric overweight and obesity [45]. Shikimic acid has antibacterial, anti-inflammatory, hair-growth-stimulating, and anti-aging effects as well as antifungal properties [46]. Menatetrenone significantly decreases undercarboxylated osteocalcin to osteocalcin and improves lumbar bone mineral density in osteoporotic patients [47]. High 4-pyridoxic acid/pyridoxine ratio is independently associated with global cardiovascular risk [48]. Metabolomics and transcriptomic analysis highlighted that *Klebsiella oxytoca* P620 application decreases the intensities of pyridoxine and glucarate O-phosphoric acid in PHBA-stressed leaves, and downregulates the expression of genes related to these metabolites [49]; *evm.model.Contig191.1* (*RrLHY*), *evm.model.Contig100.63* (*RrREVEILLE7*); pyridoxine exhibited the same variation trend (Figure 9B), indicating that they might upregulate the expression of the genes related to pyridoxine.

The content of amino acids was significantly related to at least one in vitro antioxidant activity during “Rr-5” fruit development (Figure 1), but not related to three in vitro antioxidant activities in six *R. roxburghii* fruits [6]. Typically, amino acids have been reported as antioxidant substances. Fodor et al. reported that free amino acids contributed to FRAP [50]. Cit has the characteristics of a hydroxyl radical scavenger [51]. Glutamic acid and histidine decreased the generation of the hydroxyl radicals [52]. Hwang et al. demonstrated a strong antioxidant activity of methionine and lysine [53]. The results of Tian et al. showed that the electronic and hydrogen-bonding properties of the amino acids in the tripeptide sequences, and the steric properties of the amino acid residues at the C- and N-termini, play a major role in the antioxidant activities of the tripeptides. Tripeptides exhibit the highest FRAP to contain Cys (C) and Trp (W) residues [54]. Finally, in this study, in both the developing *R. roxburghii* “Rr-5” fruits and six different genotype fruits, two amino acid derivatives (MetO and L-lysine-butanoic acid) were confirmed to be significantly related to at least one in vitro antioxidant activity. Interestingly, MetO could not effectuate as an antioxidant. However, Met can reduce reactive oxygen species (ROS) levels through the activity of the Met sulfoxide reductase (MSR) system, in which MSRs act as natural scavenging systems for ROS by catalyzing the conversion of MetO to Met [55]. The phenomenon that the content of MetO is correlated to three in vitro antioxidant activities (Figure 9A) might be attributed to the high content of MetO stimulating the MSR system, thereby playing an indirect role in the in vitro antioxidant activities. In the current study, one of the MYBs, *evm.model.Contig144.143* (*RrRAX3*), and MetO had a similar variation trend (Figure 9B), indicating that *evm.model.Contig144.143* (*RrRAX3*) positively regulates the *MSR* gene expression and the MSR activity.

The characteristic metabolites of *R. roxburghii* were analyzed at the development stages. For Rr5-30, 59 characteristic metabolites were identified, including seven key metabolites (5-galloylshikimic acid, glucarate o-phosphoric acid, gallic acid, tercatain, 4-acetamidobutyric acid, shikimic acid, 1-linoleoylglycerol). No key metabolites were found in Rr5-60 and Rr5-90. From the perspective of antioxidation, Rr5-30 has high antioxidant properties, and can be used as a raw material for antioxidant products. The characteristic metabolites were also used to perform a query on the traditional Chinese medicine database and analysis platform (TCMSP; tcmsp-e.com, accessed on 7 August 2021). For Rr5-30, Ser and Thr were identified as medicinal components. The eight medicinal substances identified from the characteristic metabolites of Rr5-90 were GABA, Met, Val, Lys, His, Tyr, Hcy, and Arg. No medicinal components were found in Rr5-60. Reportedly, Met and Val can be used to synthesize glucose during starvation, Lys can be used to manufacture ketones as an alternative energy source for the body [56], and Arg and Thr regulate the immune response in the body [57], among which Arg has the best effect [58]. In addition, Arg can stimulate insulin and regulate human blood glucose levels [59]. From a medicinal point of view, the Rr5-90 has a significant value. We can select *R*. *roxburghii* fruit at a specific development stage for processing according to its medicinal value or antioxidant value so as to improve the utilization rate of sparse fruits in planting.

The amino acid synthesis was complex. According to the difference in the synthetic skeleton, amino acids could be divided into six categories: glutamate amino acids, aspartic acid, serine family, histidine, aromatic amino acids, and alanine. The analysis of the amino acids of the glutamate family revealed that *GPT* (*evm.model.Contig339.70*) affects GABA, Arg, and 5-AVA. *GLT1* (*evm.model.Contig386.230*) and *NAGS* (*evm.model.Contig273.43*) affect the accumulation of Cit, and *ACY1* (*evm.model.Contig290.142*) affects the accumulation of Cit, GABA, and Arg. The ACY1 enzyme catalyzes the production of ornithine from N-acetylornithine. Fremont et al. showed that *ACY1* has a significant effect on Arg accumulation [60]. Under drought stress, ornithine accumulation in watermelon increases and the expression of N-acetylornithine aminotransferase (*clcg09003180*) increases significantly [61]. These studies showed that *ACY1* was the key gene of the Arg synthesis pathway. The results showed that the upstream gene had a maximal influence on the metabolites of the glutamate family. COT1 was the first enzyme synthesized in the aspartic acid family. The high expression of *COT1* (*evm.model.Contig120.160, evm.model.Contig283.202*) leads to the overall upregulation of the aspartic acid family pathway. L-homocysteine, Met, SAH, and Lys were affected by the synthesis pathway genes, while Thr was affected by *ThrC* (*evm.model.Contig 264.74*) and *MetC* (*evm.model.Contig243.89*). *ThrC* (*evm.model.Contig264.74*) and *MetC* (*evm.model.Contig243.89*) competed for L-homoserine. The low expression of *ThrC* (*evm.model.Contig264.74*) and the high expression of *MetC* (*evm.model.Contig243.89*) decreased, while the accumulation of L-homocysteine, *Met*, and *SAH* increased the accumulation of L-(−)-threonine.

Aromatic amino acids are precursors of polyphenols. Tyr, TA, and L-phenylalanine were substrates for the synthesis of phenolic acids. Therefore, the high accumulation of Tyr and TA guaranteed the high accumulation of phenolic acids. Flavonoids and phenolic acids were the representative metabolites of *R. roxburghii*, with critical roles in the antioxidant activity of *R. roxburghii*. In this study, we elucidated the importance of 4-coumaric acid: coenzyme A ligase (4CL) (EC 6.2.1.12) to phenolic acids. 4CL synthesized cinnamic acid and its hydroxyl or methoxy derivatives, such as 4-coumaric acid, caffeic acid, ferulic acid, and 5-hydroxyferulic acid. Erucic acid was the substrate. The activity of 4CL influenced the accumulation of phenolic acids, and it was one of the major enzymes in the phenylpropanoid biosynthetic pathway [62,63]. Flavonoids were affected by many genes in this pathway, and any single gene could not be used to explain the changes in flavonoids.

Complex biochemical pathways were regulated by polygenic reactions and could not be explained by structural genes alone. Moreover, transcription factors play a critical role in biochemical pathways. The analysis of the promoter elements of structural genes identified many binding sites of the transcription factors on structural genes, especially *MYB*. Herein, we identified 158 *RrMYBs* in the transcriptome of *R. roxburghii* fruit, of which 75 showed a significant differential expression. In previous studies, 30 *CcMYBs* were involved in flavonoid and lignin biosynthesis in pigeon pea [64]. In pears, MYB was considered a key regulator affecting flavonoid biosynthesis [65]. *TaMYB-A1* also regulated anthocyanin biosynthesis in *Triticum aestivum* [66]. The MYB transcription factor exerted a significant influence on the biosynthetic pathway of plants. The correlation between metabolites, structural genes, and transcription factors was presented in a network diagram, and six *RrMYBs* were identified as the potential key regulatory factors. Lu et al. demonstrated that 15 *RrMYBs* regulated the lignin of *R. roxburghii*, among which *evm.model.Contig 191.1* (*RrLHY*) was reported by Lu et al. [11] to regulate lignin biosynthesis, further indicating *evm.model.Contig 191.1* (*RrLHY*) as the key transcription factor. Silencing *GhRAX3* reduced the ability of cotton to eliminate the hazardous effects of ROS [67]. In the present study, *evm.model.Contig144.143* (*RrRAX3*) showed a specific effect on scavenging free radicals. We also speculated that *evm.model.Contig361.14* (*RrMYB105*) and *evm.model.Contig100.63* (*RrREVEILLE7*) are key *RrMYBs* in *R. roxburghii.* Herein, four key *MYBs* play a critical role in plants. *REVEILLE7* and *LHY* are circadian clock-related genes, while *PbRVE7* upregulated the expression of *PbDFR* and *PbANS* and promoted the accumulation of anthocyanins in pear peel [68]. Recent studies have demonstrated that LHY participates in cold stress by regulating the expression of *DREB1* [69]. Zhang et al. showed that *RAX3* regulates cuticle biosynthesis and is a key regulator of trichome initiation in sand rice (*Agriophyllum squarrosum*) [70]. *OsMYB105* participates in the drought resistance of rice [71].

## 5. Conclusions

In this study, total phenols, flavonoids, triterpenoids, and 21 monomers were suggested to be key metabolites, contributing to the in vitro antioxidant activities, irrespective of the different developmental stages of fruits or in fruits of different *R. roxburghii* genotypes. Phenolic acid, flavonoid, and amino acid biosynthesis networks were constructed with 32 structural genes, 48 *RrMYBs*, and 23 metabolites by the conjoint analysis of the metabolome and transcriptome in *R. roxburghii* fruits at different development stages. Among these, four key *RrMYBs* affected the significantly varied amino acids, phenolic acids, and flavonol derivatives in the network during fruit development and the metabolites related to in vitro antioxidative activities in fruits of six *R. roxburghii* genotypes.

## Figures and Tables

**Table 1:** Bioactive substance content and in vitro antioxidant capacities during *R. roxburghii* fruit development. (Lowercase letters in the table indicate significance, and different letters indicate significant differences among the groups).

	Organic Acid (% FW)	Amino Acid (μmol/g FW)	Flavonoids (mg/100 g FW)	Total Phenols (mg/100 g FW)	Terpenoids (mg/100 g FW)	AsA Content (mg/100 g FW)	Lipids (% FW)	DPPH (μmol TE/g FW)	FRAP (μmol TE/g FW)	ABTS (μmol TE/g FW)
Rr5-30	0.58 ± 0.04 c	22.23 ± 1.96 a	2250.12 ± 139.77 a	4540.25 ± 451.04 a	7543.93 ± 39.71 a	70.78 ± 0.23 c	0.90 ± 0.07 a	78.57 ± 0.41 a	1902.78 ± 78.84 a	154.29 ± 3.89 a
Rr5-60	0.83 ± 0.07 b	9.05 ± 1.57 b	1416.51 ± 58.23 b	3430.60 ± 50.15 b	5024.89 ± 286.07 b	285.08 ± 10.12 b	0.94 ± 0.04 a	58.76 ± 3.42 c	891.67 ± 43.83 b	58.96 ± 3.46 c
Rr5-90	1.12 ± 0.04 a	6.95 + 0.94 b	456.32 + 30.86 c	2376.71 ±202.62 c	1701.63 ± 23.51 c	861.13 ± 15.56 a	1.11 ± 0.16 a	66.04 ± 3.23 b	140.97 ± 8.72 c	66.03 ± 3.21 b

## Data Availability

The metabolomic data of *R. roxburghii* ‘Guinong 5’ fruits at three different developmental stages: 30, 60, and 90 DAAs, are included in the present article. The mature fruit metabolomic data of the other five *R. roxburghii* genotypes, Rr-1, Rr-3, Rr-4, Rr-7, and Rr-f, can be found in Ref. [6]. The transcriptomic data of ‘Guinong 5’ fruits at three developmental stages: 30, 60, and 90 DAAs can be found in Ref. [11]. The RNA sequencing RAW data can be found in the Sequence Read Archive (SRA) database at NCBI (https://submit.ncbi.nlm.nih.gov/subs/sra, accessed on 20 March 2022) and are available under study accession number PRJNA533675.

## Supplementary Materials

The following supporting information can be downloaded at: https://www.mdpi.com/article/10.3390/foods11111639/s1. Figure S1: *Rosa roxburghii* fruits of three developmental stages. Scale bar = 1 cm; Figure S2: Repeated principal component analysis and correlation assessment of the samples. (**A**) Principal component analysis of metabolites. Each point in the figure represents a sample, and the sample of the same group is represented by the same color. (**B**) Analysis of the correlation between the samples; Figure S3: KEGG classification of differentially accumulating metabolites. (**A**) KEGG classification of differentially accumulating metabolites between Rr5-30 and Rr5-60. (**B**) KEGG classification of differentially accumulating metabolites between Rr5-60 and Rr5-90. (**C**) KEGG classification of differentially accumulating metabolites between Rr5-30 and Rr5-90; Figure S4: Expression analysis of 15 genes related to the amino acid, phenylpropanoid, and flavonoid biosynthesis during fruit development of R. roxburghii. UBQ was used as the internal control. The error bars represent the standard error of the three biological replicates. The numbers above the graphics correspond to values obtained with Pearson’s correlation; Table S1: The list of metabolites identified in *Rosa roxburghii* fruits; Table S2: The list of characteristic metabolites identified in each growth. Table S3: The list of DEGs identified in amino acid, phenolic acids and flavonol derivatives. (A) Flavonoid pathway; (B) Polyphenols; (C) Amino acids; Table S4: Correlation between DEGs and Characteristic Metabolitesin in amino acid, phenolic acids and flavonol derivatives. (A) Flavonoid pathway; (B) Polyphenols; (C) Amino acids; Table S5: MYB transcription factors that potentially regulate metabolites. (A) Flavonoid pathway; (B) Polyphenols pathway; (C) Amino acids pathway; Table S6: Primer sequences for qRT-PCR analysis.
