# Supplementary material for: Metabolomics Combined with Transcriptomics Analysis Revealed the Amino Acids, Phenolic Acids, and Flavonol Derivatives Biosynthesis Network in Developing Rosa roxburghii Fruit
Source: Foods. 2022 Jun 1;11(11):1639. doi: 10.3390/foods11111639 (PMC9180193; doi:10.3390/foods11111639)
Supplement: Supplementary file 1 [file foods-11-01639-s001.zip › Supplementary Figures.pdf]

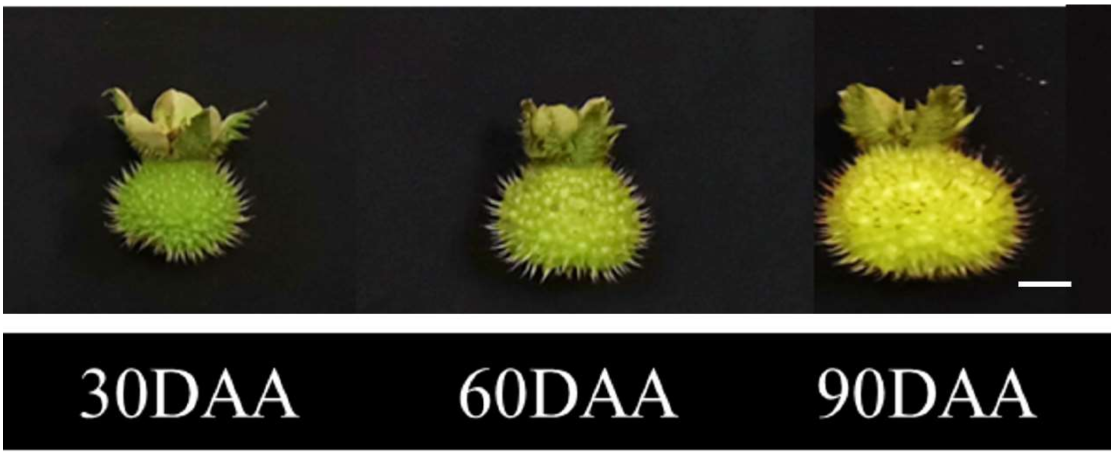

Figure S1. *Rosa roxburghii* fruits of three developmental stages. Scale bar = 1 cm

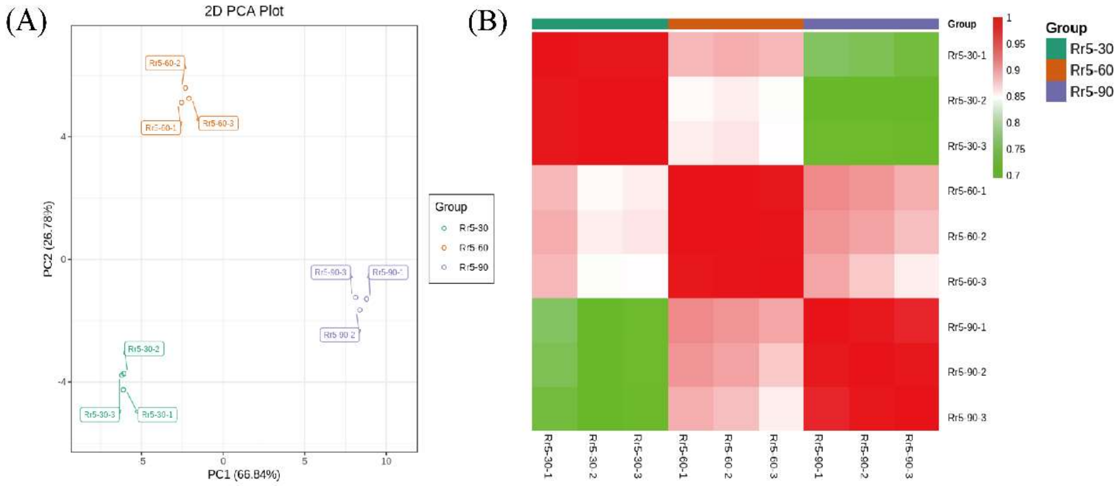

Figure S2. Repeated principal component analysis and correlation assessment of the samples. (A) Principal component analysis of metabolites. Each point in the figure represents a sample, and the sample of the same group is represented by the same color. (B) Analysis of the correlation between the samples.

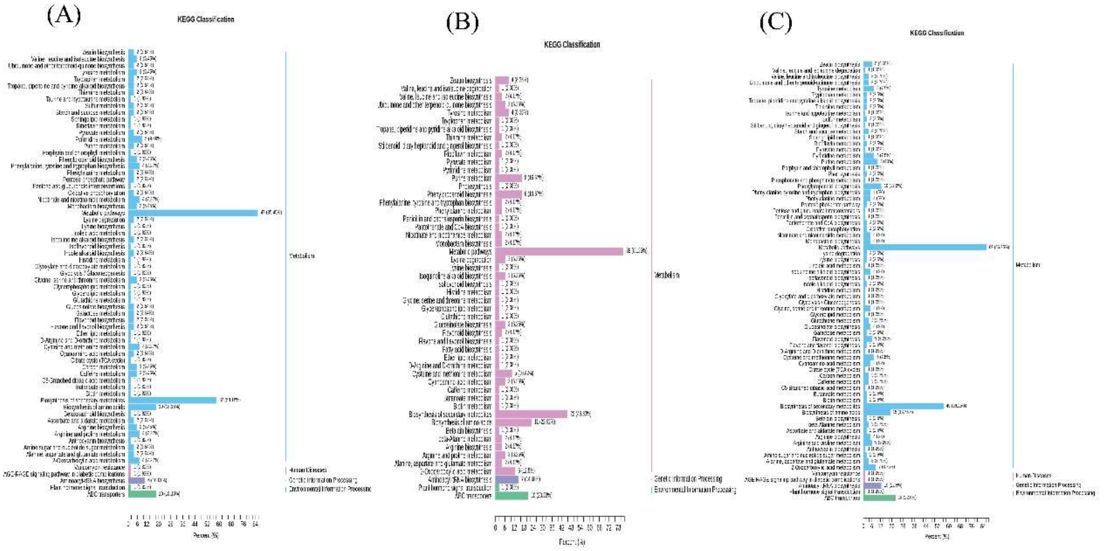

Figure S3. KEGG classification of differentially accumulating metabolites. (A) KEGG classification of differentially accumulating metabolites between Rr5-30 and Rr5-60. (B) KEGG classification of differentially accumulating metabolites between Rr5-60 and Rr5-90. (C) KEGG classification of differentially accumulating metabolites between Rr5-30 and Rr5-90.

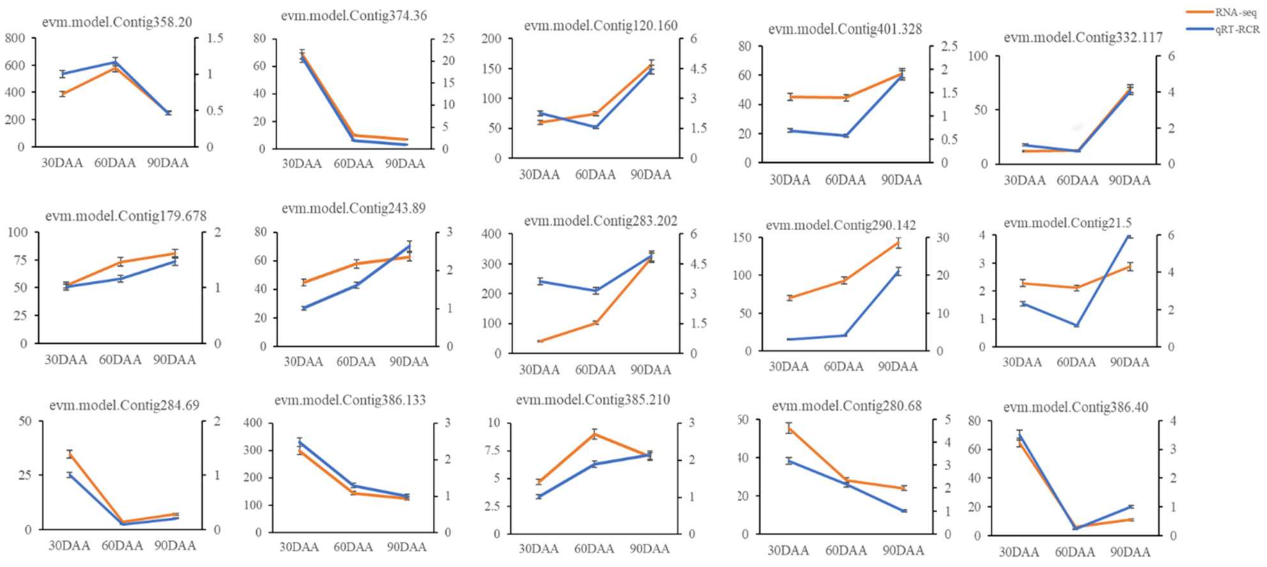

Figure S4. Expression analysis of 15 genes related to the amino acid, phenylpropanoid, and flavonoid biosynthesis during fruit development of *R. roxburghii*. UBQ was used as the internal control. The error bars represent the standard error of the three biological replicates. The numbers above the graphics correspond to values obtained with Pearson's correlation.
